# Digital death and posthumous data: a glocal, relational perspective

**DOI:** 10.3389/fgene.2026.1885207

**Published:** 2026-07-29

**Authors:** Adela Toplean

**Affiliations:** Department of Communication Sciences, Faculty of Letters, University of Bucharest, Bucharest, Romania

**Keywords:** digital afterlife, digital death, digital remains, glocalisation, personhood, posthumous data, DeathTech

## Abstract

Persistent digital presence now routinely outlives biological death through memorialized profiles, algorithmic “memories,” AI-mediated avatars, or biobank reanalysis. This perspective argues that decisions about such posthumous data—including postmortem genomic data—should be grounded in a glocally situated, relational view of personhood and practice, rather than in one-size-fits-all, individual-only rules. A small shared vocabulary is first proposed for use across disciplines: digital afterlife, digital immortality, posthumous lifespan, digital remains, and digital death. Digital death is treated phenomenologically as a lived, relational process: a hybrid online–offline experience in which dying, mourning, remembrance, and legacy unfold across digital and non-digital contexts. Second, it is argued that selves persist through relations, roles, and traces and that digital and genomic data amplify this relationality. Posthumous decisions, therefore, affect kin and communities and call for relational, kin-sensitive consent rather than static, individual authorizations. Third, a glocal lens is advanced: digital and data infrastructures always meet local death systems—institutions, rites, beliefs, and moral orders—that refract global capabilities into self-limiting “boundary solutions.” To make this usable for genetics and allied fields, a brief action triage is outlined—focusing on stakes, relational radius, local death system, and time frame—and illustrate with a posthumous variant re-analysis/return-of-results scenario to help choose the least intrusive viable pathway. While approximate, such staged, glocally attuned practice can reduce foreseeable harm and support ethically coherent, clinically workable, and culturally credible uses of posthumous data.

## Introduction

This perspective advances a glocally situated, relational governance approach to posthumous data—data about or derived from a deceased person that persists after biological death. By “glocal,” it is meant that global infrastructures and standards are refracted through local cultures and institutions (Robertson, 1995; Roudometof, 2016). This approach differs from generic contextual frameworks by locating decision authority in local death systems and by operationalizing dividual personhood through kin-sensitive stewardship and attention to timing across the posthumous lifespan (Brubaker et al., 2014; Davies, 2024; Kastenbaum, 2018; Toplean, 2024b).

Two domains of posthumous data are particularly relevant here. The first is postmortem genomic data—stored sequences and linked clinical records that remain re-analyzable with implications for biological relatives and clinical practice (Harbinja, 2019; Jarvik et al., 2014; Latimer et al., 2022). The second is the digital afterlife—the deceased’s continued social presence mediated by digital remains and, at times, AI memorial agents (Hurtado, 2026; Kasket, 2019; Savin-Baden and Mason-Robbie, 2020; Stokes, 2021). Although these domains differ, they share structural features that justify treating them together under the umbrella of “posthumous data”: both implicate kin and communities beyond the individual; both raise questions of consent and responsibility; both are shaped by local death systems; and in both, timing determines whether an intervention helps or harms (Altaratz and Morse, 2023; Brubaker et al., 2014; Harbinja, 2019).

Genetics-ELSI scholarship has already addressed key aspects of posthumous return and family implications, including ante-mortem preferences (Wolf et al., 2015), IRB variability in oversight (Beskow et al., 2015), actionability thresholds (Jarvik et al., 2014), and collective rights in genomic data (Hudson et al., 2020; H3Africa Consortium, 2018). Similarly, non-Western legal perspectives highlight the need for proportionate criteria, as observed in Romanian jurisprudence on forensic DNA databases (Nestor, 2024) and Brazil’s LGPD treatment of genetic data as sensitive (Autoridade Nacional de Proteção de Dados (ANPD), 2026; Brasil, 2018). Building on this foundation, the glocal–relational approach adds three elements that remain underdeveloped: locally authoritative “boundary solutions,” kin-sensitive stewardship grounded in dividual personhood, and explicit pacing over the posthumous lifespan.

Concretely, this perspective makes three contributions: a concise vocabulary bridging genetics and digital contexts; a relational–glocal framework centered on kinship, ritual authority, trust, and timing; and an operational action triage for clinicians, genetic counselors, biobank managers, and regulators. The three principles draw on a multi-year qualitative study of digital death in several European countries (Christensen and Sumiala, 2024; Toplean, 2023a; Toplean, 2023b; Toplean, 2024a; Toplean, 2024b; Toplean, 2024c) and serve here as a conceptual scaffold. Unless otherwise indicated, claims are grounded in published studies and this ethnographic work; where extrapolations are made, proposals are offered as testable governance hypotheses.

## Identifying gaps

Despite a rapidly expanding literature on digital remains, several conceptual and practical gaps persist. Theory remains fragmented, while platforms and services are often developed without glocally grounded frameworks or domain expertise (Christensen and Sumiala, 2024; Kasket, 2019; Savin-Baden, 2022; Sisto, 2020, 2021). This theory–practice divide, compounded by thin governance, leaves practitioners improvising around digital remains and their posthumous lifespan, often outpacing ethics and misreading local death systems (Kastenbaum, 2018; Conte and Serafini, 2026; Morse and Birnhack, 2024; Toplean, 2023a; Toplean, 2023b).

Second, timing and local compatibility are systematically under-weighted in current policy and platform design. What helps in month six can harm in week one, and technically feasible options can violate local moral economies of grief (Hurtado, 2026; Sumiala and Jacobsen, 2024; Öhman, 2024). Timing—decisive in dying, decision-making, and acute grief—is also underweighted, even as posthumous data linger and are repurposed across platforms, archives, and AI memorial agents (Kasket, 2019; Thommandru and Mone, 2026; Walter, 2017).

Third, current debates often separate “online” from “offline” and assume a bounded, autonomous individual. This leaves under-examined how contemporary experience flows across digital and non-digital contexts and how the digital afterlife is embedded in glocally situated relations of kinship, care, and moral obligation (Toplean, 2015; Toplean, 2023a; Toplean, 2023b; Toplean, 2026). Commercial logics—surveillance capitalism and expanding thanatechnology markets—further accelerate postmortem datafication faster than ethical appraisal can keep pace (Bassett, 2022; Kneese, 2023; Zuboff, 2019; Lindemann, 2022).

Fourth, existing governance frameworks remain poorly suited to posthumous data. Major regimes (e.g., GDPR Recital 27) are silent on the deceased, and genomic persistence raises kin duties and disclosure dilemmas that individual-consent models cannot resolve (Grandinetti et al., 2020; Harbinja, 2017, 2019, 2022; Miller et al., 2021; Öhman and Floridi, 2017; Öhman, 2024). A systematic review of stakeholder perspectives showed that the majority of biobanks lack explicit policies on the post-mortem use of genetic and health-related data, creating significant uncertainty after participants’ death (Bak et al., 2020). Research involving samples from deceased individuals, such as in molecular autopsy, also raises complex consent and privacy issues, with suicide decedent data occupying an ethical gray area regarding next-of-kin authorization and secondary research use (Shade et al., 2019).

## A shared vocabulary and three framing principles

This section offers a concise vocabulary and three framing principles that shift attention from technologies to the ways posthumous data operate within relationships, local time frames, and meaning systems across intertwined online–offline contexts. These concepts provide a cross-disciplinary foundation for understanding the complex social and ethical challenges surrounding posthumous data.

### Vocabulary

Digital afterlife refers to the deceased’s continued presence and actions mediated by digital remains, platform curation, and AI memorial agents, without implying duration (Savin-Baden and Mason-Robbie, 2020). Digital immortality is an imaginary of indefinite persistence that typically ignores data fragility and platform/commercial contingencies; it is a culturally embedded rather than universal horizon, with imaginaries differing markedly across religious, cultural, and political contexts (Hurtado, 2023; Nowaczyk-Basińska et al., 2025; Park, 2020; Sutherland, 2023; Nowaczyk-Basińska and Kiel, 2024; Toplean, 2023b). Posthumous lifespan refers to the durability and impact of posthumous data. Digital remains are the artifacts and derivatives (content, metadata, biometrics, and model outputs) that can be recombined or repurposed. Digital death usually refers to the processes through which dying, mourning, remembrance, and legacy become networked, vernacular, and platformed (Sumiala and Jacobsen, 2024; Pitsillides et al., 2013; Sofka, 2020; Sisto, 2020). These are not merely technical descriptors but culturally and morally charged categories. For some communities, genomic data are collectively held; stewardship should reflect communal rights and expectations (Hudson et al., 2020).

### Framing principles

#### Phenomenology of digital death

Contemporary encounters with mortality are inherently hybrid: offline embodied relations and online interactions co-produce how dying is managed and how loss is felt, narrated, and sustained (Toplean, 2023b). It is argued that digital death is best understood as a lived sociocultural experience of mortality across online and offline contexts. For genetics and related fields, this backdrop is crucial for understanding posthumous data-use decisions on re-analysis, return, and reuse, which must be calibrated across persons, time frames, contexts, and platforms.

#### Dividual personhood and relational consent

In the digital age, “dividuality transcends the ‘individual’” (Davies, 2024). Selves persist through relations, roles, and data (Harju, 2024; Roudometof, 2023). Therefore, stewardship extends to kin and communities, favoring kin-sensitive, relational consent over purely individual, static permissions (Brubaker et al., 2014; Davies, 2017; Davies, 2024; Gergen, 2011; Rosa, 2019; Kastenbaum, 2018; Harbinja, 2017; Öhman and Floridi, 2017; Toplean, 2015; Toplean, 2024c; Toplean, 2026).

#### Glocal boundary solutions

Global infrastructures are refracted through local death systems (institutions, rites, authorities, privacy norms, and moral values), yielding self-limiting, context-sensitive ways of using or resisting technologies (Robertson, 1995; Roudometof, 2016; Roudometof and Dessi, 2022; Walter, 2012; Ohnuki-Tierney et al., 1994; Toplean, 2024a; Toplean, 2024b; Toplean, 2026). It is argued elsewhere that four traits of digital glocalization of mortality recur: adoption is self-limiting; kin and ritual authority outrank platform affordances (relational primacy); tools create techno-affective spaces without displacing metaphysics; and change emerges from communities rather than from above (Toplean, 2023a; Toplean, 2024a).

## From principles to practice: a short action triage

To make this glocal, relational stance usable, this perspective proposes a minimal decision aid. Unlike existing family-based and tiered-timing frameworks from major professional bodies (e.g., ACMG SF v3.0; ESHG recontacting recommendations), this triage makes timing and local cultural compatibility explicit operational criteria. It requires a designated family steward, a least-intrusive default during crisis windows, and documented deferral with a timed review (Carrieri et al., 2019; Miller et al., 2021).

Practitioners should (1) specify the intervention and stakes—what is being proposed and are the primary stakes related to health, reputation, or ritual?; (2) map the relational radius—which kin and communities are implicated, acknowledging dividual personhood and network effects?; (3) characterize the local death system—rites, authorities, trust and privacy norms, stigma, and media sensitivities; and (4) determine temporality—whether the context involves end-of-life or acute bereavement or later commemorative phases. Where possible, identify a family steward—a kin-endorsed liaison who coordinates contact, consent-to-contact, and pacing—and note red-flag incompatibilities (e.g., prohibitions on naming, livestreaming, or simulation).

After answering (1)–(4), (5) select a pathway: (a) proceed with safeguards; (b) modify or scope down; or (c) defer or refrain; and (6) document the rationale, communicate with stakeholders, and set a review point aligned with the posthumous lifespan.

Role cues. Clinicians and genetic counselors should align with local recontact policies, use consent-to-contact through a family steward, and offer counseling before disclosure. Platform designers should feature-gate high-intensity tools (e.g., AI memorial agents), default to private or small-audience modes, and provide clear opt-out and sunset controls (Thommandru and Mone, 2026).

These steps implement the relational–glocal approach by co-deciding with locally authoritative actors and pacing decisions over the posthumous lifespan. If the timeframe is acute or local fit is uncertain, the default is minimal intervention: private, kin-led practices and deferral of high-intensity tools and public spectacle. Where stakes are high and incompatibility is evident, it may be preferable to refrain, document the reasons, and set a review date.

While not claiming that this framework would outperform existing guidance in every case, it is argued that it offers a supplementary lens by making timing and local cultural compatibility explicit operational criteria. Consider a scenario in which a laboratory re-interprets a pathogenic variant in a deceased woman, with implications for first-degree relatives. Given the high health stakes, the relational radius (adult children and siblings), a conservative religious family’s distrust of hospitals, and the 3-year timeframe, the triage would prompt the clinical team to first approach a designated family steward or local ethics body rather than sending unsolicited notifications to all kin. In a more recent death or a different cultural setting, the same questions could justify deferring contact or narrowing disclosure.

The above steps are necessarily approximate. Digital remains grow, leak, and recombine, and many social, psychological, and legal effects remain unknown (Cann, 2014; Stokes, 2021). Even so, staged, kin-sensitive practice can reduce foreseeable harm by pacing re-analysis and return-to-grief timeframes, working through a family steward with counseling support, and defaulting to a pause when uncertainty or local incompatibility is high.

## Conclusion

This perspective argued that the governance of posthumous genomic and digital data requires more than individual consent or platform policy. A glocal, relational lens shows how these data are refracted through local death systems and why culturally thin models often fail in practice. By clarifying key terms, re-specifying personhood as dividual, and offering an operational action triage, this perspective provides decision-makers in genetics and digital ethics with a practical tool to pace interventions, involve kin, and respect local norms—supporting posthumous practice that is ethically coherent, psychologically safe, clinically workable, and culturally legitimate.

## Data Availability

The original contributions presented in the study are included in the article/supplementary material; further inquiries can be directed to the corresponding author.
